# Quantitative Prediction of Solvation Free Energy in Octanol of Organic Compounds

**DOI:** 10.3390/ijms10031031

**Published:** 2009-03-11

**Authors:** Eduardo J. Delgado, Gonzalo A. Jaña

**Affiliations:** Theoretical and Computational Chemistry Group (QTC), Faculty of Chemical Sciences, Casilla 160-C, Universidad de Concepción, Concepción, Chile; E-Mail: gjana@udec.cl

**Keywords:** QSPR, solvation free energy, octanol, organic compounds

## Abstract

The free energy of solvation, $\Delta G_{S}^{0}$, in octanol of organic compunds is quantitatively predicted from the molecular structure. The model, involving only three molecular descriptors, is obtained by multiple linear regression analysis from a data set of 147 compounds containing diverse organic functions, namely, halogenated and non-halogenated alkanes, alkenes, alkynes, aromatics, alcohols, aldehydes, ketones, amines, ethers and esters; covering a $\Delta G_{S}^{0}$ range from about −50 to 0 kJ·mol^−1^. The model predicts the free energy of solvation with a squared correlation coefficient of 0.93 and a standard deviation, 2.4 kJ·mol^−1^, just marginally larger than the generally accepted value of experimental uncertainty. The involved molecular descriptors have definite physical meaning corresponding to the different intermolecular interactions occurring in the bulk liquid phase. The model is validated with an external set of 36 compounds not included in the training set.

## 1. Introduction

Octanol is a straight chain fatty alcohol with eight carbon atoms and molecular formula CH_3_(CH_2_)_7_OH which is used as a good surrogate for the lipids in aquatic and animal biota, and for the organic matter in soils and sediments. Lipophilicity is widely recognized as one of the key physicochemical descriptors used to assess and model the distribution and transport potential of pollutants in biological and environmental compartments, such as solubility in water, octanol-water partitioning, bioconcentration in aquatic organisms, and soil or sediment sorption phenomena [1,2]. The free energy of solvation in octanol may be used as a measure of the lipophilic nature of chemicals, i.e., as a key parameter to quantify their lipophilicity.

Gibbs free energy is arguably the most important general concept in physical chemistry, as it determines equilibrium constants and the direction of spontaneous chemical changes at constant T and P. The most theoretical work defines the standard-state free energy of solvation, $\Delta G_{S}^{0}$, as the free energy change involved in the transfer of 1 mol of solute from the gas phase into certain solvent, taking as standard state a concentration 1 mol L^−1^ in both the gas phase and solution. In addition to its fundamental interest, $\Delta G_{S}^{0}$ may be combined with other thermodynamic data to predict a variety of equilibrium properties, the more important of which are solubility and the partitioning of a solute between immiscibles phases; for instance, the calculation octanol-air partition coefficient and the octanol-water partition coefficient requires of reliable values of the free energy of solvation in octanol. The above coefficients are the critical importance in drug design, extractions and environmental applications [3].

The best developed classical method to compute free energies of solvation is the use of multivariate quantitative structure-property relationship (QSPR) models. These methods can be classified into two broad categories, namely group contribution methods, which capitalize on the additive-constitutive nature of $\Delta G_{S}^{0}$, and regression methods, which quantify the weights of different structure descriptors on the principle of least square deviation [4].

The main criticism of QSPR methods relies on the difficulty of the physical interpretation of the descriptors involved in the correlation equations; however, even within this limitation QSPR modeling represents a good alternative for rapid estimation of a property, since it requires both less computational requirements, hardware and software, and much less computation time in comparison to quantum mechanical and discrete methods (MD and MC simulations) [5,6].

Although for octanol related partition coefficients (octanol/water or octanol/air partition coefficients) there are numerous QSPR studies reported in literature; the direct prediction of free energy of solvation in octanol, by QSPR methodology, has been much less studied preventing to have a simple model for the rapid and direct estimation of $\Delta G_{S}^{0}$. Currently, free energy of solvation is estimated from some derived parameter, such as the Ostwald solubility or octanol/water partition coefficient [7].

In this article, we report a simple QSPR model for predicting free energy of solvation in octanol of 147 structurally different organic compounds containing diverse organic functions. The model, involving only three molecular descriptors, allows the prediction of $\Delta G_{S}^{0}$ with a square correlation coefficient, R^2^, of 0.93 and a root-mean-square deviation of 2.41. The model is validated with an external set of 36 compounds not included in the training set.

## 2. Experimental

### 2.1. Chemical data

The data set of free energy of solvation in octanol used to develop the model was taken from the data reported by Kollman *et al.* [8]. The data set contains 147 structurally different compounds containing diverse organic functions: alkanes, alkenes, alkynes, aromatics, alcohols, aldehydes, ethers, ketones, esters, amines, nitriles, halogenated and nitro hydrocarbons covering a $\Delta G_{S}^{0}$ range from about – 50 to 0 kJ/mol. The data for the validation set was collected from several literature sources [9–11].

### 2.2. Computational methodology

Initial three-dimensional geometries for all 147 chemicals in their ground state were generated using the Hyperchem 7.0 molecular modeling package [12]. Subsequent quantum chemical calculations in gas phase were performed using the AMPAC program [13] with AMI parameterization. The output files of AMPAC containing the refined geometries and electron wave functions of individual compounds were loaded into the CODESSA program [14] in order to calculate a total of 453 molecular descriptors. This pool of descriptors was reduced by objective feature selection in order to remove those descriptors that do not provide useful information for the prediction of $\Delta G_{S}^{0}$. Pairwise correlations between descriptors were examined so that only one descriptor was retained from a pair contributing similar information. Finally, with this reduced pool of descriptors the best multiple linear regression model was searched with SigmaStat [15] by fitting the descriptors to the experimental data and respective correlation analysis.

## 3. Results and Discussion

In this study, a total of 453 molecular descriptors were calculated for all 147 compounds. The molecular descriptors can be grouped as constitutional, geometric, topological, electrostatic, and quantum chemical. However, bearing in mind that the main interactions between uncharged molecules are originated by polar, dispersion and hydrogen-bonding interactions; we have focused on descriptors encoding those features, and in this way the number of potential descriptors useful for the prediction of $\Delta G_{S}^{0}$ was reduced to 119.

The best multiple linear regression model found involves only the following three molecular descriptors: the gravitation index (G_I_), HA dependent HDCA-2, and the number of fluorine atoms (N_F_); which will be defined further on. The resulting equation is:

(1) $$\Delta \bar{G_{S}^{0}}\;=\;0.46\;-\;0.033G_{I}\;-\;29.77HA\;+\;4.09N_{F}$$

and its respective statistics is shown in Tables 1 and 2. The statistical parameters are the following: R the correlation coefficient, R_CV_ the leave-one-out crossvalidated correlation coefficient, R_df_ the correlation coefficient adjusted for degrees of freedom, s the standard deviation, F the Fisher test value, P the probability to retain the null hypothesis, i.e. the independent variables have no predictive value and therefore the observed relationship occurred just by chance; and VTF, the variation inflation factor, is a measurement of the collinearity between the independent variables. If the VIF value is 1, there is no collinearity. If its value is large, about 10 or more, serious collinearity is present.

The squared correlation coefficient value, R^2^, indicates that the model as fitted explains 93% of the variability of the property. The squared crossvalidated correlation coefficient, R^2^_CV_, provides an estimation of the stability of the obtained regression model, i.e. the sensitivity of the model to the elimination of any single data point. For the model the value of this parameter is equal to the squared correlation coefficient value indicating a good stability of the regression model. The correlation coefficient adjusted for degrees of freedom is a useful figure for comparing models with different numbers of independent variables. In determining whether the model can be simplified, all the P-values on the independent variables are less than 0.001, which means that all descriptors are statistically significant at the 99% confidence level. Therefore, it is concluded that all the independent variables included in the model are relevant. The standardized coefficients represent the change in response for a change of one standard deviation in a predictor. The use of these standardized coefficients removes the problem of the predictor's underlying scale of units. On the other hand, the VIF values, equal to 1, confirm the orthogonality of the involved descriptors.

The P-value for the correlation is less than 0.001, therefore there is a statistically significant relationship between the property and the independent variables at the 99% confidence level. However, it is also observed from this Table that the value of the constant is less than the respective error and the respective P-value to retain the null hypothesis is quite large, indicating the constant has no additional predictive value over and above that contributed by the independent variables. On the other hand, the generally accepted value of experimental uncertainty for the free energy of solvation is about 1.7 kJ·mol^−1^ [16], value greater than the value of the constant. For all these reasons the constant can be eliminated from the model without loss of predictive quality and without need of refitting because the orthogonality of the involved descriptors assure the correlation coefficients and statistics remain the same. Therefore, the model is reduced to:

(2) $$\Delta \bar{G_{S}^{0}}\;=\;-0.033G_{I}\;-\;29.77\;HA\;+\;4.09\;N_{F}$$

**Figure 1.**
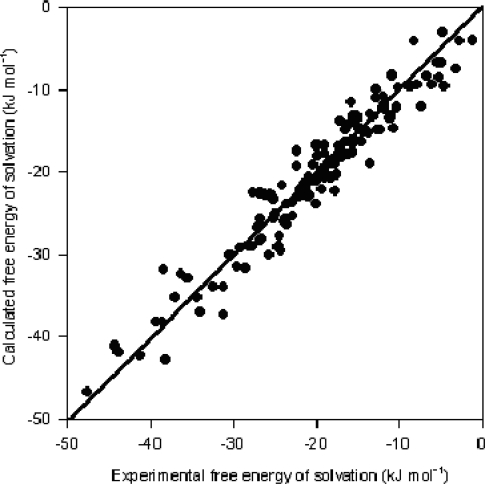
Scatter plot of calculated *vs* experimental $\Delta G_{S}^{0}$ values.

The experimental and calculated values of $\Delta G_{S}^{0}$ along with the values of the descriptors involved in the model are shown in Table 3. The respective scatter plot is shown in Figure 1.

The last column column of this Table shows the standardized residuals, i.e., the residuals expressed in standard deviation units. These residuals are used in order to better detect unusual observations. In general, if the residuals are normally distributed about the regression, about 66% of the standardized residuals have values between −1 and +1, and about 95% of the standardized residuals have values between −2 and +2. A larger standardized residual indicates that the point is far from the regression; the suggested value flagged as an outlier is 2.5. In this study, only 8 observations out of the 147 data points of the training set have standardized residual values greater than 2, of which only 3 have values over 2.5.

To check the predictive capability of the model, it was tested with an external set of chemicals not included in the training set. The validation data set included 36 diverse chemicals, including fluorotelomer alcohols and sulfur containing compounds. In Table 4, the values of the molecular descriptors along with the experimental and calculated values of $\Delta G_{S}^{0}$ for the validation set are shown. The statistics for the validation is as follows: R^2^ = 0.93, s = 2.5, F= 153. For this data, only two observations have standardized residual values greater than the threshold value of 2.5 These results confirm the prediction capability of the model.

### 3.1. Physical meaning of the descriptors involved

The more relevant descriptor in the correlation is the gravitation index (all bonds), G_I_, accounting for about the 52% of the variability of the property. This descriptor reflects the effective mass distribution in the molecule and it is defined as:

$$G_{I}\;=\;\sum_{i,j}\frac{m_{i}m_{j}}{r_{ij}^{2}}$$

where *m_i_*, and *m_j_* are the atomic masses of the bonded atoms and *r_ij_* denotes the respective bond lengths, has been associated to size-dependent bulk effects, dispersion and cavity formation, in the bulk liquid media [17]. The correlation coefficient for this descriptor is negative, as expected, indicating the solvation process is favored by these effects, leading to lower $\Delta G_{S}^{0}$ values.

The second more relevant descriptor is the hydrogen acceptor dependent hydrogen donors charged surface area based on Zefirov charges (HA dependent HDCA-2) [18]:

$$HDCA\;-\;2\;=\;\sum_{D}\frac{q_{D}\sqrt{S_{D}}}{\sqrt{S_{tot}}}\;\;\;;\;D\;\in \;H_{H-donor}$$

where S_D_ is the solvent accessible surface area of H-bonding donor H atoms, *q_D_* is the partial charge on H-bonding donor H atoms, and S_tot_ is the total solvent accessible molecular surface area. This descriptor, which accounts for about the 25% of the variability of the $\Delta G_{S}^{0}$, is connected with the hydrogen-bonding ability of the molecule. It is expected the solvation will be favored, more negative value of $\Delta G_{S}^{0}$, by the presence of hydrogen bonding interactions between solute and solvent molecules. The negative value of the coefficient for this descriptor corroborates this assumption.

Lastly, the third descriptor is the number of F atoms (N_F_). The introduction of substituents into organic compounds with increasing differences in electronegativity with respect to carbon produces a charge separation in the bond originating a dipole moment. Therefore this descriptor encodes information related to polar interactions. The question which remain is, why the molecular dipole moment then is not involved in the model ?. The answer is apparently for the deficiency of the AM1 method to calculate partial charges in molecules containing fluorine atoms. As result, the N_F_ appears to be a better descriptor for the quantitative prediction of $\Delta G_{S}^{0}$. Finally, since this descriptor encodes information relative to the polarity of the molecule, it is expected this descriptor disfavors the solvation process in octanol, a paradigm of hydrophobic solvent. Consequently, the correlation coefficient for this descriptor is positive showing the $\Delta G_{S}^{0}$ increases its value as the number of fluorine atoms also increases.

## 4. Conclusions

The merit of the QSPR model developed in this article lays in its simplicity. The model allows the prediction of $\Delta G_{S}^{0}$ of a wider variety of organics compounds with less parameters and better statistics than other models reported in literature [7]. Free energy of solvation can be predicted straightforwardly from only three molecular descriptors accounting for the different components which comprise the free energy of solvation: electrostatic, cavitation and van der Waals components. The three involved descriptors can be calculated solely from the molecular structure, therefore the model is independent of previous group contribution fitting and consequently is applicable to new or developing compounds for which group contribution has not been determined.

## Figures and Tables

**Table 1.** Statistical parameters for the best QSPR model.

	Coefficien t	Std. Error	P-value	t-test	Std. Coeff.	VIF
**Constant**	0.46	0.55	0.401	0.86		
**G_I_**	−0.033	8.96x10^−4^	< 0.001	−36.91	−0.81	1.02
**HA**	−29.77	1.11	< 0.001	−26.75	−0.58	1.03
**N_F_**	4.09	0.25	< 0.001	16.37	0.36	1.05
R^2^ = 0.93, R^2^_CV_ = 0.93, R^2^_df_ = 0.93, s = 2.41						

**Table 2.** Analysis of variance of the best QSPR model.

Analysis of Variance					
	DF	SS	MS	F	P
**Regression**	3	11700	3900	670	< 0.001
**Residual**	143	832	5.82		
**Total**	146	12533	85.84		

**Table 3.** Molecular descriptors and values of experimental and calculated $\Delta G_{S}^{0}$ for the training set.

Name	G_I_	HA dependent HDCA-2	N_F_	$\Delta G_{S}^{0}$ exp. (kJ·mol^−1^)	$\Delta G_{S}^{0}$ calc. (kJ·mol^−1^)	Standardized residual
**Alkanes**						
Ethane	122.30	0.00	0	−2.68	−4.04	0.56
Propane	204.48	0.00	0	−5.27	−6.75	0.61
Cyclopropane	251.65	0.00	0	−6.69	−8.30	0.67
2-Methylpropane	285.71	0.00	0	−6.07	−9.43	1.39
2,2-Dimethylpropane	366.02	0.00	0	−7.28	−12.08	1.99
*n*-Butane	286.70	0.00	0	−7.78	−9.46	0.70
Cyclopentane	407.86	0.00	0	−11.09	−13.46	0.98
*n*-Pentane	368.90	0.00	0	−10.25	−12.17	0.80
*n*-Hexane	451.10	0.00	0	−12.59	−14.89	0.95
Cyclohexane	492.57	0.00	0	−14.48	−16.25	0.74
Methylcyclohexane	574.01	0.00	0	−13.43	−18.94	2.29
*n*-Heptane	533.30	0.00	0	−15.65	−17.60	0.81
*n*-Octane	615.51	0.00	0	−17.49	−20.31	1.17
Chlorotrifluoromethane	495.97	0.00	3	−8.24	−4.10	−1.72
Dichlorodifluoromethane	503.91	0.00	2	−5.23	−8.45	1.34
Fluorotrichloromethane	521.24	0.00	1	−11.00	−13.11	0.88
1,1,2-Trichloro-1,2,2-trifluoroethane	818.05	0.00	3	−10.63	−14.73	1.70
1-Bromo-1-chloro-2,2,2-trifluoroethane	832.16	0.00	3	−13.68	−15.19	0.63
**Alkanes**						
Bromotrifluoromethane	596.15	0.00	3	−3.14	−7.40	1.77
Dichloromethane	300.40	0.00	0	−12.84	−9.91	−1.21
Trichloromethane	427.79	0.00	0	−15.94	−14.12	−0.76
Chloroethane	250.24	0.00	0	−10.79	−8.26	−1.05
1,1,1 -Trichloroethane	501.12	0.00	0	−15.44	−16.54	0.46
1,1 -Difluoroethane	340.12	0.00	2	−4.73	−3.04	−0.70
1,1,2-Trichloroethane	507.54	0.00	0	−18.95	−16.75	−0.91
1-Chloropropane	332.45	0.00	0	−12.80	−10.97	−0.76
2-Chloropropane	329.56	0.00	0	−11.88	−10.88	−0.42
Bromomethane	293.94	0.00	0	−10.17	−9.70	−0.20
Dibromomethane	550.46	0.00	0	−17.49	−18.17	0.28
Tribromomethane	801.73	0.00	0	−23.51	−26.46	1.22
Bromoethane	370.65	0.00	0	−12.13	−12.23	0.04
2-Bromopropane	453.05	0.00	0	−14.23	−14.95	0.30
1-Bromobutane	535.20	0.00	0	−17.41	−17.66	0.10
1-Bromopentane	617.39	0.00	0	−19.58	−20.37	0.33
Nitroethane	495.74	0.00	0	−16.44	−16.36	−0.03
1-Nitropropane	577.83	0.00	0	−18.58	−19.07	0.20
2-Nitropropane	575.60	0.00	0	−17.70	−18.99	0.54
1-Nitrobutane	659.99	0.00	0	−21.38	−21.78	0.17
**Alkenes**						
Ethylene	122.21	0.00	0	−1.13	−4.03	1.20
Propylene	206.54	0.00	0	−4.77	−6.82	0.85
2-Methylpropene	290.01	0.00	0	−8.49	−9.57	0.45
1-Butene	288.64	0.00	0	−4.56	−9.53	2.06
1-Hexene	453.03	0.00	0	−12.30	−14.95	1.10
1,3-Butadiene	290.52	0.00	0	−8.79	−9.59	0.33
*cis-*1,2-Dichloroethylene	400.65	0.00	0	−15.52	−13.22	−0.95
*trans-*1,2-Dichloroethylene	399.06	0.00	0	−15.10	−13.17	−0.80
Trichloroethylene	537.42	0.00	0	−15.69	−17.73	0.85
Tetrachloroethylene	674.93	0.00	0	−17.74	−22.27	1.88
3-Bromopropene	455.31	0.00	0	−13.81	−15.03	0.50
**Alkynes**						
1-Pentyne	375.21	0.00	0	−11.67	−12.38	0.30
1-Hexyne	457.40	0.00	0	−14.35	−15.09	0.31
**Aromatics**						
Benzene	504.85	0.00	0	−15.56	−16.66	0.46
Toluene	588.93	0.00	0	−19.04	−19.43	0.16
Ethylbenzene	670.93	0.00	0	−21.25	−22.14	0.37
*m*-Xylene	672.96	0.00	0	−21.97	−22.21	0.10
*o*-Xylene	672.78	0.00	0	−21.21	−22.20	0.41
*p*-Xylene	673.07	0.00	0	−21.71	−22.21	0.21
Naphtalene	887.05	0.00	0	−29.16	−29.27	0.05
Anthracene	1269.00	0.00	0	−43.81	−41.88	−0.80
Bromobenzene	767.77	0.00	0	−22.84	−25.34	1.04
Fluorobenzene	617.17	0.00	1	−16.19	−16.28	0.04
1,4-Dibromobenzene	1031.60	0.00	0	−31.25	−34.04	1.16
*p*-Bromotoluene	852.06	0.00	0	−26.61	−28.12	0.63
Chlorobenzene	641.42	0.00	0	−20.92	−21.17	0.10
1,2-Dichlorobenzene	779.27	0.00	0	−25.15	−25.72	0.23
1,4-Dichlorobenzene	778.61	0.00	0	−23.72	−25.69	0.82
Nitrobenzene	879.27	0.00	0	−27.74	−29.02	0.53
2-Nitrotoluene	962.38	0.00	0	−28.45	−31.76	1.37
2,2′-Dichlorobiphenyl	1158.20	0.00	0	−39.37	−38.22	−0.48
2,3 -Dichlorobiphenyl	1160.50	0.00	0	−38.62	−38.30	−0.13
2,2,3′-Trichlorobiphenyl	1295.30	0.00	0	−38.16	−42.74	1.90
**Alcohols**						
Methanol	142.96	0.44	0	−16.19	−17.82	0.67
Ethanol	224.20	0.44	0	−18.24	−20.50	0.94
Ethylene glycol	326.09	0.89	0	−31.13	−37.26	2.54
1-Propanol	306.49	0.43	0	−21.00	−22.92	0.79
2-Propanol	305.16	0.40	0	−19.33	−21.98	1.10
1,1,1 -Trifluoro-2-propanol	638.10	0.43	3	−21.42	−21.59	0.07
Hexafluoro-2-propanol	972.10	0.47	6	−24.10	−21.53	−1.07
1-Butanol	388.67	0.45	0	−23.89	−26.22	0.97
*tert*-Butyl alcohol	385.36	0.38	0	−20.00	−24.03	1.67
1-Pentanol	470.88	0.43	0	−26.78	−28.34	0.65
1-Hexanol	553.07	0.45	0	−29.54	−31.65	0.87
1-Heptanol	635.29	0.44	0	−32.43	−34.06	0.68
1-Octanol	717.49	0.45	0	−34.02	−37.07	1.27
1-Decanol	881.86	0.45	0	−41.34	−42.50	0.48
Allyl alcohol	308.24	0.43	0	−22.05	−22.97	0.38
Phenol	612.17	0.41	0	−36.36	−32.41	−1.64
4-Bromophenol	875.94	0.41	0	−44.31	−41.11	−1.33
2-Cresol	695.97	0.34	0	−35.52	−33.09	−1.01
3-Cresol	696.13	0.41	0	−34.31	−35.18	0.36
4-Cresol	696.29	0.41	0	−36.99	−35.18	−0.75
2,2,2-Trifluoroethanol	557.11	0.50	3	−20.13	−21.00	0.36
2-Methoxyethanol	432.48	0.45	0	−24.39	−27.67	1.36
**Ethers**						
Methyl propyl ether	413.02	0.00	0	−15.19	−13.63	−0.65
Methyl isopropyl ether	411.69	0.00	0	−15.19	−13.59	−0.67
Methyl tert-butyl ether	492.04	0.00	0	−15.19	−16.24	0.43
Diethyl ether	412.05	0.00	0	−15.19	−13.60	−0.66
Tetrahydrofuran	451.51	0.00	0	−16.44	−14.90	−0.64
Anisole	718.19	0.00	0	−22.89	−23.70	0.34
Ethyl phenyl ether	799.40	0.00	0	−23.64	−26.38	1.14
1,2-Dimethoxyethane	538.90	0.00	0	−19.04	−17.78	−0.52
1,4-Dioxane	580.19	0.00	0	−20.46	−19.15	−0.55
l,l-Dichloro-2,2-difluoroethylmethyl ether	800.57	0.00	2	−16.82	−18.24	0.59
**Aldehydes**						
Formaldehyde	147.19	0.27	0	−13.51	−12.90	−0.26
Propanal	312.73	0.22	0	−17.28	−16.87	−0.17
Butanal	394.91	0.22	0	−19.33	−19.58	0.10
Benzaldehyde	696.53	0.24	0	−25.65	−30.13	1.86
*m*-Hydroxybenzaldehyde	803.86	0.68	0	−47.66	−46.77	−0.37
*p*-Hydroxybenzaldehyde	804.41	0.64	0	−51.71	−45.60	−2.54
**Ketones**						
Acetone	312.77	0.10	0	−13.18	−13.30	0.05
2-Butanone	395.36	0.10	0	−15.82	−16.02	0.08
3,3-Dimethylbutanone	554.92	0.08	0	−18.95	−20.69	0.72
2-Pentanone	476.64	0.10	0	−18.20	−18.71	0.21
3-Pentanone	476.00	0.11	0	−18.24	−18.98	0.31
Cyclopentanone	518.01	0.11	0	−20.96	−20.37	−0.25
2-Hexanone	559.88	0.09	0	−21.00	−21.16	0.06
**Ketones**						
2-Heptanone	642.08	0.09	0	−23.64	−23.87	0.09
2-Octanonne	724.29	0.09	0	−26.69	−26.58	−0.05
Acetophenone	778.46	0.11	0	−28.20	−28.96	0.32
**Esters**						
Methyl formate	363.42	0.00	0	−11.80	−11.99	0.08
Methyl acetate	446.71	0.00	0	−14.81	−14.74	−0.03
Ethyl acetate	527.89	0.00	0	−16.99	−17.42	0.18
Propyl acetate	610.15	0.00	0	−19.04	−20.13	0.45
Butyl acetate	692.32	0.00	0	−20.75	−22.85	0.87
Methyl propionate	528.62	0.00	0	−16.99	−17.44	0.19
Methyl butyrate	605.56	0.00	0	−19.20	−19.98	0.33
Methyl pentanoate	692.80	0.00	0	−21.46	−22.86	0.58
Methyl benzoate	911.96	0.00	0	−30.38	−30.09	−0.12
**Amines**						
Methylamine	139.01	0.23	0	−15.82	−11.43	−1.82
Ethylamine	219.44	0.22	0	−17.11	−13.79	−1.38
Propylamine	301.77	0.23	0	−19.96	−16.81	−1.31
Butylamine	383.96	0.22	0	−22.38	−19.22	−1.31
Diethylamine	395.38	0.17	0	−19.87	−18.11	−0.73
Dipropylamine	559.65	0.16	0	−25.19	−23.23	−0.81
Trimethylamine	328.18	0.09	0	−15.06	−13.51	−0.64
**Miscellaneous**						
Piperazine	544.97	0.39	0	−24.27	−29.59	2.21
Aniline	609.22	0.22	0	−27.11	−26.65	−0.19
Morpholine	562.85	0.22	0	−25.06	−25.12	0.03
Piperidine	518.83	0.19	0	−26.23	−22.78	−1.43
Pyridine	529.10	0.00	0	−22.34	−17.46	−2.02
2-Methylpyridine	611.55	0.09	0	−25.69	−22.86	−1.17
3-Methylpyridine	613.61	0.08	0	−26.78	−22.63	−1.72
4-Methylpyridine	613.30	0.08	0	−27.61	−22.62	−2.07
2-Ethylpyridine	693.46	0.09	0	−26.78	−25.56	−0.50
2-Methylpyrazine	717.78	0.18	0	−24.56	−29.05	1.86
Benzonitrile	689.18	0.00	0	−25.48	−22.74	−1.14
2,6-Dichlorobenzonitrile	963.97	0.00	0	−38.41	−31.81	−2.74

**Table 4.** Molecular descriptors and values of experimental and calculated $\Delta G_{S}^{0}$ for the validation set.

Name	G_I_	HA dependent HDCA-2	N_F_	$\Delta G_{S}^{0}$ exp. (kJ·nnol^−1^)	$\Delta G_{S}^{0}$ calc. (kJ·mol^−1^)	Standardized residual
1,2,3 -Trichlorobenzene	917.09	0.00	0	−30.36	−30.26	−0.04
1,2,4-Trichlorobenzene	916.84	0.00	0	−29.10	−30.26	0.46
1,2,3,4-Tetrachloro-benzene	1055.20	0.00	0	−33.21	−34.82	0.64
Pentachlorobenzene	1193.60	0.00	0	−37.09	−39.39	0.92
1,2,3 -Trichloronaphthalene	1297.90	0.00	0	−43.48	−42.83	−0.26
Methane	39.19	0.00	0	2.13	−1.29	1.37
Chloromethane	169.87	0.00	0	−7.42	−5.61	−0.73
Tetrachloromethane	549.75	0.00	0	−14.49	−18.14	1.46
Monochloroethane	250.23	0.00	0	−9.87	−8.26	−0.64
1,1 -Dichloroethane	377.46	0.00	0	−14.61	−12.46	−0.86
1,1,1,2-Tetrachloroethane	632.35	0.00	0	−22.65	−20.87	−0.71
Chloropropane	332.47	0.00	0	−13.18	−10.97	−0.88
1,2,3-Trichloropropane	589.65	0.00	0	−25.79	−19.46	−2.53
4:2 FTOH	1537.40	0.46	9	−26.08	−27.62	0.62
6:2 FTOH	2136.40	0.46	13	−27.50	−31.03	1.41
8:2 FTOH	2735.40	0.46	17	−31.84	−34.43	1.04
10:12 FTOH	3334.50	0.46	21	−32.58	−37.84	2.11
12:2 FTOH	3933.60	0.46	25	−35.38	−41.25	2.35
Ethyl formate	444.76	0.00	0	−12.50	−14.68	0.87
Propyl formate	526.87	0.00	0	−15.18	−17.39	0.88
Isopentanol	469.57	0.45	0	−25.79	−28.89	1.24
Cyclohexanol	593.62	0.39	0	−29.56	−31.20	0.66
Hexanal	559.31	0.22	0	−25.16	−25.01	−0.06
4-Methyl-2-pentanone	558.35	0.09	0	−18.83	−21.10	0.91
Ethyl propionate	610.06	0.00	0	−17.97	−20.13	0.86
Isopropyl acetate	608.75	0.00	0	−18.09	−20.09	0.80
Di-isopropyl ether	573.56	0.00	0	−15.18	−18.93	1.50
Ethyl butyrate	692.21	0.00	0	−20.31	−22.84	1.01
Pentafluorobenzene	1070.60	0.00	5	−14.49	−14.88	0.16
Hexafluorobenzene	1184.70	0.00	6	−12.04	−14.56	1.01
1-Propanethiol	335.70	0.19	0	−14.73	−16.73	0.80
Thiophenol	646.51	0.18	0	−25.06	−26.69	0.65
Thioanisole	783.48	0.10	0	−27.07	−28.83	0.70
Dimethyl sulfide	309.60	0.10	0	−17.74	−13.19	−1.82
Diethyl sulfide	470.87	0.09	0	−17.11	−18.22	0.44
Dipropyl sulfide	635.15	0.08	0	−16.28	−23.34	2.82
